# The power of phages: revolutionizing cancer treatment

**DOI:** 10.3389/fonc.2023.1290296

**Published:** 2023-11-15

**Authors:** Md. Sharifull Islam, Jie Fan, Fan Pan

**Affiliations:** ^1^ Center for Cancer Immunology, Institute of Biomedicine and Biotechnology, Shenzhen Institute of Advanced Technology, Chinese Academy of Sciences, Shenzhen, China; ^2^ Department of Cardiology, Handan Central Hospital, Handan, Hebei, China

**Keywords:** phages, cancer therapy, phage display, immunotherapy, targeted nanocarriers, vaccine development, personalized medicine

## Abstract

Cancer is a devastating disease with a high global mortality rate and is projected to increase further in the coming years. Current treatment options, such as chemotherapy and radiation therapy, have limitations including side effects, variable effectiveness, high costs, and limited availability. There is a growing need for alternative treatments that can target cancer cells specifically with fewer side effects. Phages, that infect bacteria but not eukaryotic cells, have emerged as promising cancer therapeutics due to their unique properties, including specificity and ease of genetic modification. Engineered phages can transform cancer treatment by targeting cancer cells while sparing healthy ones. Phages exhibit versatility as nanocarriers, capable of delivering therapeutic agents like gene therapy, immunotherapy, and vaccines. Phages are extensively used in vaccine development, with filamentous, tailed, and icosahedral phages explored for different antigen expression possibilities. Engineered filamentous phages bring benefits such as built in adjuvant properties, cost-effectiveness, versatility in multivalent formulations, feasibility of oral administration, and stability. Phage-based vaccines stimulate the innate immune system by engaging pattern recognition receptors on antigen-presenting cells, enhancing phage peptide antigen presentation to B-cells and T-cells. This review presents recent phage therapy advances and challenges in cancer therapy, exploring its versatile tools and vaccine potential.

## Introduction

Cancer is a multifaceted and devastating disease considered by the abnormal growth and spread of cells in the body. Globally, it is a significant contributor to mortality, causing approximately 9.6 million deaths each year, 1 in every 6 deaths is caused by this disease. Adopting a wholesome life-style lowers the chance of most cancers development via 30–50% (1). The projected global cancer burden for the year 2040 is estimated to reach 28.4 million cases, indicating a substantial 47% increase compared to the year 2020 (2). Cancer is a pathological condition that can manifest in various anatomical locations within the human body, such as lung cancer, breast cancer, prostate cancer, and colorectal cancer, among others (3). There are many risk factors that are correlated in increasing a person’s likelihood of developing cancer, including age, genetics, lifestyle factors such as tobacco and alcohol use, and exposure to environmental toxins. However, not all cases of cancer can be attributed to known risk factors, and some of individuals may progress cancer without any detectible cause. The diagnosis and treatment of cancer is complex, and the best approach will depend on the type and stage of the disease. Common treatment choices encompass chemotherapy, radiation therapy, targeted therapy, immunotherapy and surgery. The current treatment options for cancer are often constrained by various factors, such as side effects, limited effectiveness, high costs, increased risks, and unavailability in certain regions or countries (4–9). There is a growing need for alternative treatments that can target cancer cells more specifically and with fewer or no side effects. Ongoing research and development in the field of medical will hopefully lead to new and better treatment options for patients in the future. Phage therapy had a number of advantages as a potential cancer treatment. Firstly, phages are highly specific and could be designed with target cancer cells while leaving healthy cells untouched. This is in contrast to traditional cancer treatments which damage healthy tissues and organs in addition to cancerous cells. Secondly, phages are able to replicate within their host cells, which can increase the efficacy of treatment by targeting multiple cancer cells with a single phage. Finally, phages have the potential to be personalized to the individual patient by selecting or designing phages that target the specific mutations or genetic abnormalities present in the patient’s cancer cells.

Bacteriophages, often known as phages, are a wide variety of viruses having various dimensions and forms (icosahedral, like λ, T4, T7, or; filamentous, like fd, f1, M13) which can infect bacteria and lyse host bacteria to produce progeny phages for further infection, but they typically do not infect eukaryotic cells. Phages are the most predominant class of living things in the biosphere and infect almost all of the existing pathogenic bacteria (10). Two different researchers separately discovered phages: Twort in 1915 and d’Herelle in 1917 (11). Twort, a 37 years old English doctor, was trying to culture vaccinia virus, the main element in the smallpox vaccine, on agar plates. But the only things developing on Twort’s petri-dishes were contaminating bacteria, not vaccinia. Even though he wasn’t making any headway toward his initial goal, Twort noticed that something else was going on: sporadically, on his plates, strange “glassy and translucent” areas appeared that, upon closer inspection, revealed out to be clear zones on the lawn of bacteria. Twort appeared to support the theory that it was a bacterial enzyme instead of a distinct type of life. He gave up research on phages and spent his professional career developing animal viruses. A French-Canadian microbiologist named Felix d’Herelle independently published similar findings two years later and he was immediately declared a new virus type that infected bacteria, that he named as phage (12). The estimated number of phages in existence is approximately 10^31^–10^32^, they are the most prevalent biological species on Earth and are essential in controlling bacterial populations (13). When a phage infects a bacterial cell, it initiates either a lytic cycle that causes bacterial lysis or a cycle of lysogeny that unable to lyse the bacterial cells (14, 15). Usually, phages bind to particular receptors on the surface of the host cell, introduce their genetic material, and subsequently replicate either by integrating to the bacterial genome and passing vertically to daughter cells or by utilizing the bacterial replication mechanism to generate the next generation of phage progeny (16). Within few minutes of infection, phages destroy target bacteria by lysis, releasing freshly produced phage virions, which then infect new host bacteria into a self-replicating cycle (17).

Anti-cancer or anti-tumor is the new field of phage application. Phage study has entered its 2nd generation in recent times. Phages used in cancer treatment and diagnosis as targeted nanocarriers, as well as in gene therapy as vehicles for therapeutic DNAs or RNAs. Phages are studied in the sense of immunogenic vaccine study due to its capacity to trigger both cell mediated and antibody mediated responses (18). By gene insertion, the targeted proteins or peptides could be expressed in the phage capsid, presenting them to immunological compartments to trigger a strong immunological response against diverse antigens from cancerous cells (19). Due to its tiny and uniform size, it is the most effective nanoparticle for drug administration as well as for a number of other uses, such as phage display and targeting. Because they are found everywhere in nature, their capsids can be employed to induce immune response or, through genetic or protein engineering, enable any organism to express desired proteins on its surface and elicit an immune response (20). Another approach is to use phage display technology to generate recombinant humanized monoclonal antibodies that target cancer cells and infectious microorganisms (21). The M13 phage display technique was enhanced for human antibody production in the late 1980s and early 1990s through collaborative efforts from three research groups: the Scripps Research Institute in La Jolla, USA; the MRC laboratory of molecular biology in Cambridge, UK; and the DKFZ in Heidelberg, Germany (22–25). Usually, peptides are determined to be tumor targeting compounds using phage display (26). Phage DNA and phage display vaccines are two different ways that phages can be used to deliver immunizations (27). Antigens are expressed on the phage surface in vaccines through phage display. Phage display technology is responsible for phages’ major contribution to vaccine design. This strategy makes use of the intrinsic qualities of these particles, including their adjuvant potential, affordable manufacture, and optimum stability, among other things (28). Phages are safe to utilize as delivery systems for human cancer vaccines because they are unable to replicate in eukaryotic cells (29).

Phages, owing to their nanoscale dimensions, adaptable surface properties, precise target specificity, inherent safety profile, and non-pathogenic nature, exhibit substantial promise for applications in theragnostics, gene therapy, and immunotherapy in cancer treatment. The aim of this review paper is to highlight the potential of phage therapy as a revolutionary and innovative approach in the field of cancer treatment. The manuscript discusses the unique properties of bacteriophages that make them promising candidates for targeted and personalized cancer therapies. It also explores their applications as nanocarriers for drug delivery and vaccines, emphasizing the potential of phages in activating the immune response against cancer cells. The manuscript emphasizes the need for further research, clinical trials, and collaboration between scientific and medical communities to fully realize the benefits of phage therapy and its transformative impact on cancer treatment and healthcare.

### Cancer treatments

There are many different conventional approaches to treat cancer, which is a complicated disease. Conventional cancer treatment techniques generally involve a combination of surgery, radiation therapy, chemotherapy, immunotherapy, drug delivery agents and gene therapy (30). These treatment strategies have been used for ages and have been shown to be effective for controlling of cancer. Surgery is frequently the first choice of cancer treatment. It implicates the removal of cancerous tissue from the body. The primary goal of surgery is to remove as much of the cancer as feasible and prevent its spread to other organs in the body. Various types of surgery are performed based on the type and location of the cancer. For example, a lumpectomy involves removing the tumor along with a portion of surrounding tissue, while a mastectomy involves the complete removal of the entire breast (31, 32). Surgery is frequently combined with other treatments, including as chemotherapy and radiation therapy, to improve its success (33). Chemotherapy is a treatment using diverse drugs to kill cancer cells. These drugs can be administered orally or through intravenous means. Chemotherapy drugs can also affect normal cells, that’s why patients may had massive side effects like nausea, hair loss, and fatigue (34). Chemotherapy may serve as the main treatment for certain cancers or as an adjuvant therapy, employed after surgery to eliminate any remaining cancer cells. Radiation therapy, on the other hand, uses high-energy radiation to eliminate cancer cells. It can be provided outside or internal part of the body. External radiation therapy employs a machine expose ray to the specific site of cancer, whereas internal radiation therapy contains insertion a source of radioactive inside the path of body. The radiation therapy mechanism involves inflicting damage the DNA of malignant cells, hindering their ability to proliferate (33). Similar to chemotherapy, radiation therapy can lead to side effects such as skin irritation and fatigue (35). Radiation therapy can be employed alone or in conjunction with surgery and chemotherapy. The conventional cancer treatment techniques like surgery, chemotherapy, and radiation therapy have been the cornerstone of cancer treatment for many years. They have been shown to be effective in treating cancer and improving patient outcomes having different advantages and disadvantages (**Table 1**).

**Table 1:** Cancer treatment techniques with advantages and disadvantages.

Serial No	Techniques	Advantages	Disadvantages	Reference
1	Surgery	Can remove tumor entirely, immediate effect	Can be invasive, may damage nearby healthy tissue, can cause metastatic cancer cells	(36)
2	Chemotherapy	Kills rapidly dividing cancer cells, systemic effect	Can also harm healthy cells, can cause nausea, hair loss, and other side effects	(34)
3	Radiation therapy	Damages DNA of cancer cells, precise targeting	Can also harm healthy cells, can cause fatigue, skin irritation, and other side effects	(35)
4	Immunotherapy	Enhances the immune system’s ability to fight cancer, potentially long-lasting effect	Can cause immune-related side effects, may not work for all types of cancer, antibody may be less stable	(37)
5	Targeted therapy	Targets specific molecules involved in cancer growth, potentially less toxic to healthy cells	Can be expensive, If a patient’s cancer does not have specific mutations, targeted therapy may not be effective.	(38)
6	Hormone therapy	Blocks or removes hormones that fuel certain types of cancer, potentially fewer side effects than chemotherapy	May not work for all types of cancer, can cause menopausal symptoms and other side effects	(39)
7	Stem cell transplant	Replaces damaged bone marrow with healthy stem cells, potentially curative for certain types of cancer	Can be associated with significant side effects, can be difficult to find a matching donor	(40)
8	Palliative care	Relieves symptoms and improves quality of life for patients with advanced cancer, can be provided alongside other treatments	Not curative, does not treat the underlying cancer	(41)

Phages can be used to protect cancer patients against germs in addition to being a potential anticancer treatment. Recently, there has been an increasing interest in using phages as a potential therapy for cancer due to their ability to specifically target and kill cancer cells. Here are some potential benefits of using phages in cancer therapy. Specificity; phages can be engineered to specifically target cancer cells while leaving healthy cells unharmed. This is because each phage has a specific receptor that it can recognize and bind to on the surface of its target cell. Such selectivity can enhance the therapy’s efficacy while reducing potential side effects. Versatility; phages can be genetically engineered to carry payloads such as toxins or genes that can enhance their ability to kill cancer cells. They can also be utilized in conjunction with other cancer treatments like chemotherapy and radiation therapy to boost their overall effectiveness. Regarding safety, phages are generally considered safe since they do not infect human cells and have been employed for decades in the food industry to prevent bacterial contamination. In contrast to antibiotics that may raise the emergence of antibiotic-resistant bacteria, phages can evolve and adjust to effectively counter changes in the targeted bacteria. This means that they may be less likely to develop resistance and could potentially be used to treat bacterial infections that are resistant to antibiotics. Cost effectiveness; phages can be produced relatively cheaply and can be stored for long periods of time, making them a potentially cost-effective option for cancer therapy. Reduced Toxicity: phages can potentially reduce the toxicity of cancer therapy. As a result, this approach has the potential to lower adverse effects linked to conventional cancer treatments like chemotherapy and radiation therapy. Ability to penetrate tumor microenvironment; phages can penetrate the dense tumor microenvironment, which can be a significant barrier to other cancer therapies. This can potentially increase the effectiveness of the therapy and improve outcomes for cancer patients. Personalization; phages can be tailored to target specific types of cancer cells or specific mutations within cancer cells. This level of personalization can potentially increase the effectiveness of the therapy and improve outcomes for cancer patients. Potential for immunomodulation; phages may have the potential to modulate the immune system by promoting the release of cytokines and activating immune cells. Although the application of phages in cancer therapy is still at its early developmental phase, the potential advantages indicate that they may evolve into a crucial tool in the battle against cancer.

## Application of phage in cancer therapy

The concept of using phages in cancer treatment is not a recent one. Back in the early 20th century, phages were applied to treat bacterial infections related to cancer, such as gangrene and sepsis (42). However, However, with the advent of antibiotics, interest in the clinical use of phages reduced. In recent years, interest in phage therapy has been raised due to the emergence of antibiotic-resistant bacteria. By altering their surface proteins, phages can be designed to preferentially target cancer cells (43). This specificity reduces the likelihood of off-target effects and toxicity. Additionally, phages can be designed to carry payloads, such as cytotoxic agents or immunomodulatory molecules, which can enhance their efficacy in killing cancer cells and activating the immune system (44). Several studies have shown the promising potential of phages in cancer therapy. When the AGKGTPSLETTP motif from a 12-mer M13-displayed phage library was used in combination with doxorubicin (DOX), it exhibited potent anti-cancer effects in mice bearing hepatocarcinoma (HCC) tumors (45). In another study published in 2021, researchers developed a phage-based therapy for glioblastoma, a type of brain cancer, and demonstrated its ability to induce tumor regression in mice (46). Additionally, various libraries cloned in multiple phage vectors employed in *in vivo* phage display experiments.

**Figure 1:**
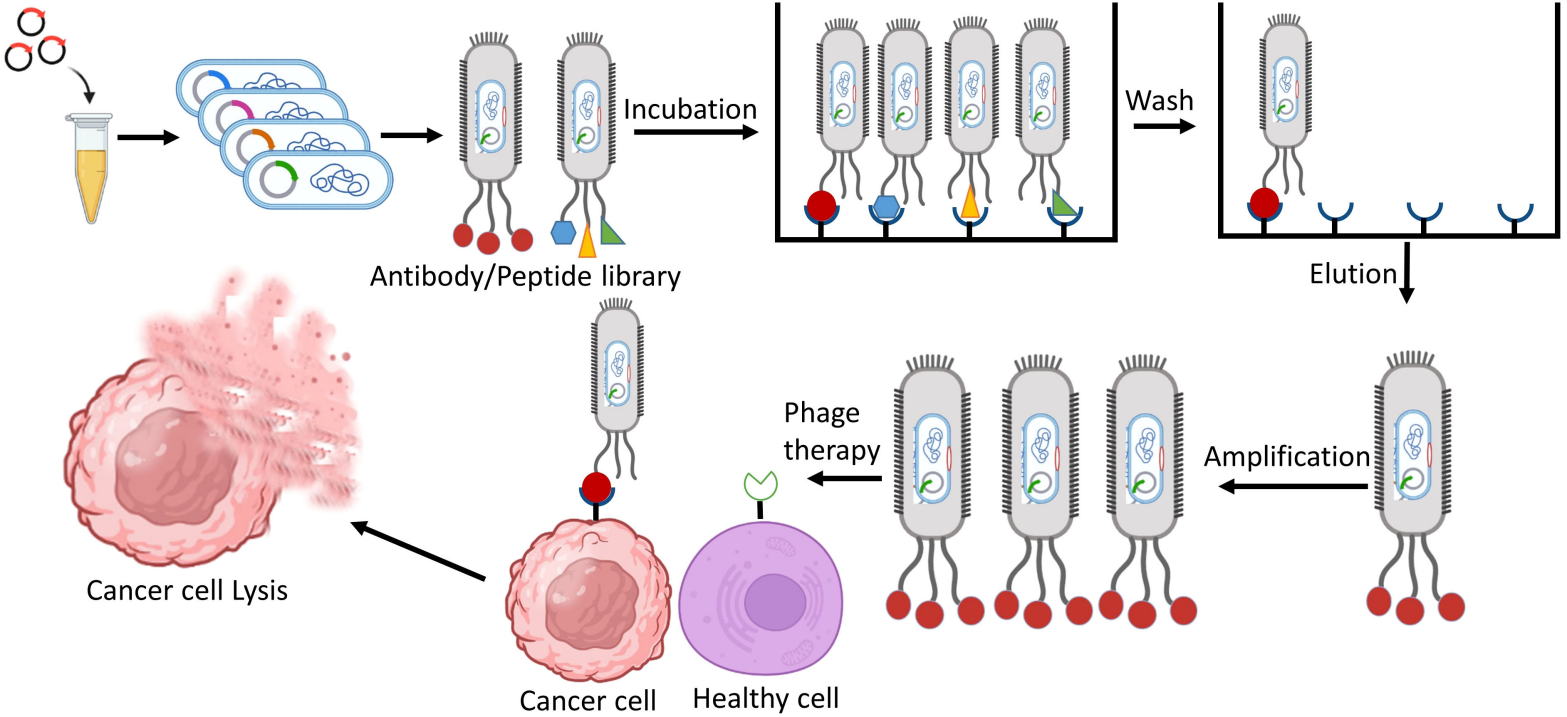
Identification of phage-displayed antibodies/peptides that specifically target cancer cells, aiming to utilize them for cancer therapy.

The process of aiming an explicit cell using a phage vector involves several sequential steps. Firstly, a screening of peptide phage library is incubated with the target of interest. This allows the phages in the library to interact with the target and bind to it. After the incubation, three to five washing steps are carried out to remove any unbound phages from the mixture. This ensures that only the phages that could successfully bound to the target cells. The next step involves the recovery of target bound phages. These phages are isolated and separated from the unbound mixture, allowing for further analysis and characterization. To amplify the target bound phages, they are introduced into bacterial cells through infection. The bacterial cells act as hosts for the phages, enabling their replication and production in larger quantities. Finally, DNA sequencing is used to identify the phage clones having the highest affinity for the target cells. The ultimate goal of targeting cancer cells using this phage display approach is to achieve cancer cell lysis. Once the phages carrying the peptides that bind to target receptors on the cancer cell surface are identified, they can be engineered or modified to deliver cytotoxic agents or induce immune responses, leading to the destruction of the cancer cells (**Figure 1**).

Phage display, a powerful technique, comprises the expression of a coat protein on the surface of a phage, leading to the presentation of a peptide. This method has found extensive applications in *in vivo* study using phage display, where multiple libraries are cloned into various phage vectors. These libraries can encompass an impressive billion clones, offering a vast diversity of sequences. In the cancer research, phage display has been employed to identify single chain antibodies capable of specifically targeting cancer cell surface antigens. These antitumor monoclonal antibodies have exhibited promising clinical potential as agents for directing therapies towards cancer cells. Today, phage display peptide libraries have become influential in the pursuit of peptide-based cell ligands, particularly those targeting cancer cells. These peptides, which are unique to specific cells and acquired through phage display systems, present significant benefits such as cost reduction, enhanced cell targeting capabilities, and a decreased chance of triggering an immune reaction, in contrast to monoclonal antibodies (47). Tumor cells commonly exhibit distinct cell surface antigens that are linked to or exclusive to the tumor. This characteristic makes them potential targets for peptide display technologies with phage, which could be engineered to selectively bind with high affinity to these peptides on the malignant cells’ surface (48). Phages can be chemically conjugated with cytotoxic medicines, increasing their therapeutic potential (49). Among the various phages used as drug delivery systems, filamentous bacteriophages stand out as one of the most valuable options. They possess the ability to carry more than hundreds drug molecules per phage and exploit the process of intracellular internalization to deliver these drugs to target cells effectively (50). For example, the M13 phage has demonstrated the capability to traverse the gastrointestinal mucosal barrier, regardless of whether a peptide is displayed on its surface. This feature opens up the possibility of orally delivering drug loaded phages, specifically targeting pathogens or specific cell types within the body (51).

Dynamic immunization against cancer antigens is an emerging strategy within cancer immunotherapy. One promising method gaining favor is the application of phages, which can display numerous proteins on their surface. Extensive research employing this technique has demonstrated encouraging outcomes in murine models, exhibiting significant reductions in tumor growth rates (52). By leveraging phage particles, the vaccine can be efficiently introduced to antigen-presenting cells, making them an ideal vehicle for vaccine delivery. Moreover, phage particles possess inherent immunogenic properties, thereby serving as a natural adjuvant (53). Phages can influence the innate immune system via influencing phagocytosis and cytokine responses. Innate immune response is induced by the naturally antigenic coat proteins of the phage head and the CpG islands in the phage genome. They can also influence adaptive immunity by altering antibody production, T helper cell activation, and the direction of effector responses (54). Naturally occurring phages have been shown to stimulate antibody formation against themselves; phage neutralizing antibodies were discovered in human serum following phage exposure (55). The spleen, one of the RES organs, is vital in the generation of anti-phage antibodies (56). Because related phages share antigens, antibodies produced during antigen presentation and stimulation can cross-react with related phages (57). Peptide-based phage display libraries can be broadly categorized into libraries of natural and random peptide (58). Notably, in several investigations, researchers used phage libraries to identify peptides those are selectively produced into blood vessels of tumor (59). An essential requirement in this field is site-specific genome integration, which offers the advantage of avoiding the complications associated with random integration while ensuring stable gene transfer. Using phage display technology, recombinant antibody fragments were established to selectively target specific subpopulations of breast cancer cells. A technology was employed to directly select these antibody fragments using tissue units. This scientific approach has the potential to uncover novel biomarkers of clinical significance when studying the antibody fragment, its binding affinity to the targeted subpopulation, and related antigens (60). In a separate study investigating the anticancer potential of phages in primary animal models, the researchers explored the effects of purified preparations of phage T4 on tumor size reduction. The results demonstrated a significant dose-dependent reduction in tumor size upon treatment. Interestingly, another phage, HAP1, exhibited even greater efficacy compared to T4, also in a dose-dependent manner (61). **Table 2** presents a comprehensive overview of the different phages employed in cancer therapy by various research groups. The information presented in this table provides valuable insights into the wide range of phages used in cancer therapy and the diverse effects they have on tumor growth.

**Table 2:** The phages for cancer therapy.

Phage	Target	Cell line	References
M13	Colorectal cancer	CT26	(62)
T4	Colon carcinoma	MC38	(63)
EFA1	Colorectal cancer	HCT-116	(64)
T7	Anti-tumor model	B16F10	(65)
M13	ovarian cancer	SKOV3 and COV362	(66)
M13	Colorectal cancer	MC38 and MC38-CEA	(67)
M13	Breast cancer	BT-474	(68)
HK022	Lung cancer	LLC-Kat, HEK293, and BJ	(69)
ANGRPSMT	Lung cancer	Calu-3	(70)
VNGRAEAP	Lung cancer	Calu-3	(70)
M13	Prostate Cancer	LNCaP	(71)
T4	Prostate Cancer	PC3	(72)
M13	Prostate Cancer	PC3	(72)
P1 and P2	Colorectal cancer	HT29, HCT116, and CT26	(73)
M13	Breast cancer	Murine fibroblast	(74)

## Bacteriophages in solid tumor theragnostics

**Figure 2:**
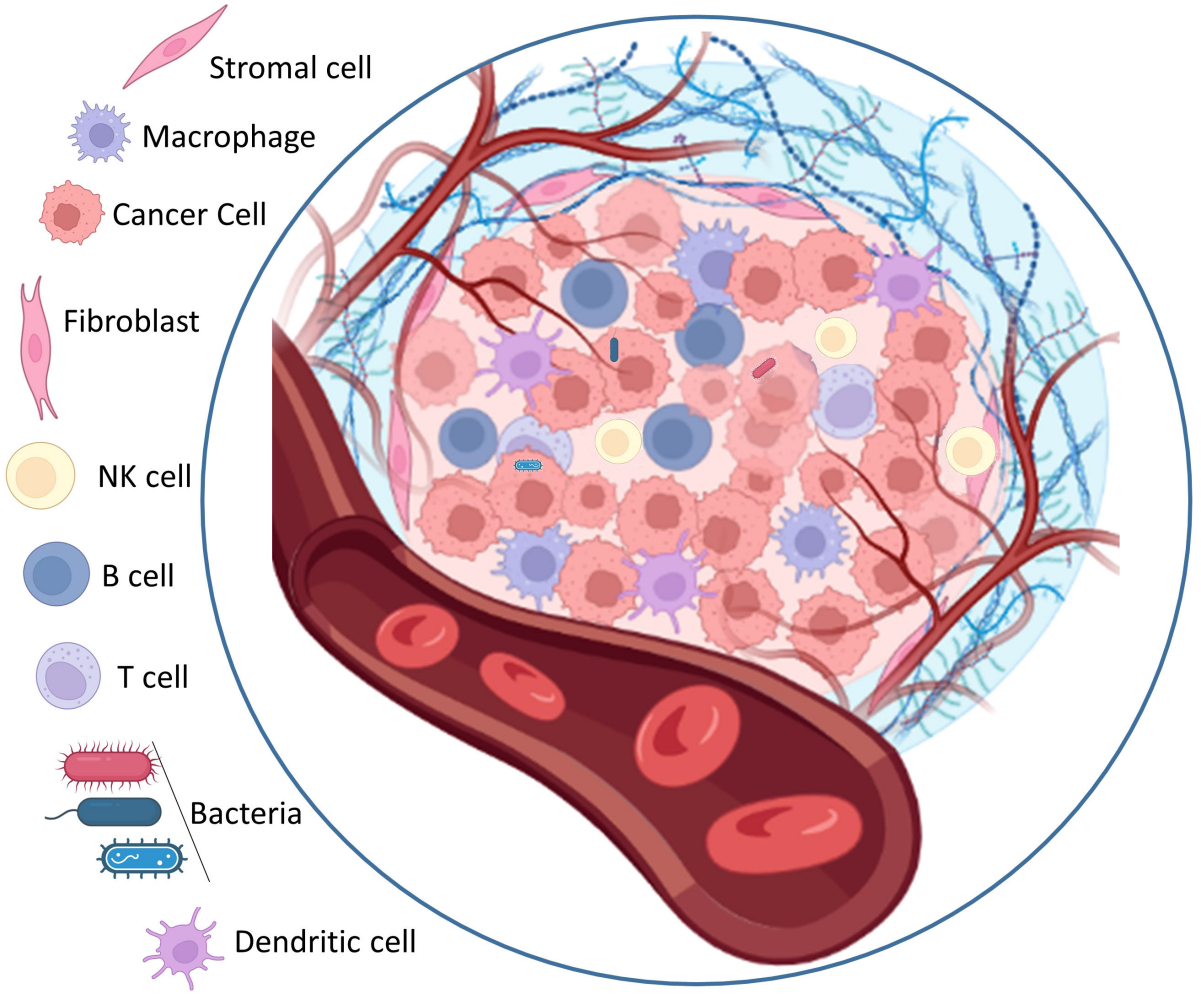
The architectural structure of solid tumor microenvironments.

Cancer is a complex disease characterized by the uncontrolled growth and proliferation of cells that differ significantly from the surrounding normal tissue. This abnormal growth occurs within a specific environment known as the tumor microenvironment, which acting a key role in supporting tumor progress and impeding the access of external factors to the tumor core. The tumor microenvironment is a highly organized and complex system of physical, chemical, and biological components that collaborate to facilitate the expansion of cancer cells. Cellular heterogeneity, a key trait of the tumor microenvironment, encompasses diverse cell types such as stromal cells, cancer-associated fibroblasts (CAFs), angiogenic endothelial cells, and immune cells linked to the tumor (75). Stromal cells are non-epithelial cells that provide structural support to the tissues and organs. Within the tumor microenvironment, stromal cells play a crucial role for tumor growth and progression (76). They can secrete growth factors, cytokines, and chemokines that encourage tumor cell proliferation, invasion, and angiogenesis the process of new blood vessel formation. Stromal cells can also remodel the extracellular matrix (ECM), a non-cellular component of the microenvironment, to create an environment more conducive to tumor growth. Cancer-associated fibroblasts (CAFs) are a specific type of stromal cell that are particularly abundant within the tumor microenvironment. CAFs have been shown to promote tumor growth by secreting growth factors and ECM-modifying enzymes, which enhance the invasive potential of cancer cells (77). They also contribute to the generation of a fibrotic environment, characterized by the deposition of excessive ECM proteins, further supporting tumor progression. Angiogenic endothelial cells are responsible for the formation of new blood vessels within the tumor microenvironment. The development of an adequate blood supply is crucial for tumor growth and metastasis, as it ensures the delivery of oxygen, nutrients, and growth factors to cancer cells. Angiogenic endothelial cells are stimulated by various signaling molecules released by tumor cells and stromal cells, promoting the sprouting and formation of new blood vessels (78). Tumor associated immune cells also play a crucial role in the tumor microenvironment. These immune cells include various types of lymphocytes, macrophages, and myeloid derived suppressor cells, among others. The interplay between tumor cells and immune cells within the microenvironment can have complex effects. While some immune cells can recognize and eliminate cancer cells, others can be coopted by the tumor to promote its growth and evade the immune response, leading to an immunosuppressive environment (79). In addition to the cellular components, the tumor microenvironment is influenced by various non cellular factors. The extracellular matrix (ECM) provides structural support to cells and can undergo dynamic changes in composition and organization within the tumor microenvironment. These alterations in the ECM can promote tumor cell migration, invasion, and metastasis. Matrix metalloproteinases (MMPs) are enzymes involved in ECM degradation and remodeling, and their dysregulation within the microenvironment can contribute to tumor progression (80). Acidosis, a condition characterized by low extracellular pH, is a common feature of solid tumors. Hypoxia, a state of low oxygen availability, often occurs due to inefficient blood supply in rapidly growing tumors. These hypoxic conditions can trigger a variety of cellular responses, including the activation of signaling pathways that promote tumor cell survival and angiogenesis (81). Overall, the tumor microenvironment forms an intricate and dynamic ecosystem consisting of diverse cellular components (**Figure 2**).

Phages, have emerged as promising theragnostic options in the field of biomedical research and applications. Their unique properties, including their nanosize, non-pathogenic nature, polyvalent surface properties, and versatility for chemical or genetic modifications, make them attractive for both therapeutic and diagnostic purposes in solid tumor (82). Phages are essentially composed of repetitive units of the same coat protein, making them uniform biologics. Their production is relatively easy as they can be prepared using bacterial hosts, which adds to their convenience as research tools (83). The macromolecular protein heads of phages offer an excellent platform for attaching diverse cargoes, such as drugs and fluorescent probes, enabling them to act as carriers for precise targeted delivery (14). Due to their small size, phages can penetrate tumors more effectively, improving drug delivery to cancerous cells (84). This characteristic also enables them to evade clearance by the reticulo-endothelial system (RES), spleen, kidney, and hepatobiliary system, prolonging their circulation in the body and enhancing their potential therapeutic efficacy (43). Moreover, phages can be personalized in solid tumor treatment by incorporating drugs, drug cocktails, targeting ligands, antagonists, and other therapeutic agents. This flexibility makes them versatile multifunctional entities that can be tailored to specific treatment strategies and individual patient needs. Phages also possess the ability to cross biological barriers that often pose challenges in drug delivery. For example, they can traverse the blood brain barrier, which is crucial for the treatment of brain tumors (85). Similarly, they can penetrate the hypovascular fibrotic barrier, enabling effective delivery of therapeutics to pancreatic tumor, which is known for its challenging microenvironment (86). The carriers possess a significant advantage in efficiently transporting therapeutic payloads to cancer cells compared to macromolecular chemotherapeutics, owing to their elevated surface to volume ratio (87). Consequently, phages have the potential to enhance the efficacy of drug delivery and improve the overall therapeutic outcome (88).

## Gene therapy for cancer utilizing bacteriophages as carriers

Phage mediated cancer gene therapy is a method that utilizes the potential of phages to specifically deliver therapeutic genes to cancer cells. Phages have been extensively studied and utilized in various fields, including medicine (89). In traditional gene therapy, viral vectors derived from mammalian viruses are commonly used to deliver therapeutic genes into target cells. However, the use of mammalian viruses can pose safety concerns and limitations due to their potential for integration into the host genome, which may lead to surprising consequences. Phages offer a promising alternative as they are highly specific to bacterial hosts and do not infect human cells (90). In the 1940s, Bloch presented the initial evidence of phages’ capacity to directly interrelate with mammalian cells (91). According to his findings, phages can aggregate in malignant cells for tissue and impede tumor development. Later, Kantoch demonstrated that phages could attach to guinea pig leukocytes and penetrate them. Recent research has additionally supported the regular interactions between phages and mammalian immune cells (92). Merril conducted tests in the 1970s that established the lambda phage’s ability to interact with human fibroblast cells (93). The concept behind phage based cancer gene therapy is to engineer these phages to carry therapeutic genes and specifically target cancer cells while sparing healthy cells. This can be achieved by modifying the phage’s surface proteins or tail fibers to recognize and bind to cancer cell markers or receptors. Once the modified phages attach to the cancer cells, they can deliver the therapeutic genes into the cells (43). The therapeutic genes carried by phages can be designed to achieve various objectives in cancer treatment. For example, they can encode proteins that induce apoptosis in cancer cells, inhibit tumor developments, or enhance the immune response against cancer cells. By delivering these therapeutic genes directly to cancer cells, phage based gene therapy offers the potential for targeted and precise treatment with minimal off-target effects (94). Moreover, phages have inherent advantages as gene delivery vehicles. They are relatively easy to manipulate in the laboratory, and their ability to infect a broad range of bacteria enables the development of a diverse library of phage variants with different tumor targeting properties. Additionally, phages can be administered via various routes, including intravenous injection, local injection, or even oral administration, providing flexibility in treatment options (14). Despite the significant promise of phage based cancer gene therapy, there are still challenges to overcome. Additional investigation is necessary to refine phage design, improve targeting efficiency, and ensure the safety and effectiveness of this strategy. Ongoing clinical trials are being conducted to assess the therapeutic possibilities of phage based gene therapy for various cancer types (95).

Phage-based cancer vaccines have emerged as a highly important and promising field in modern biotechnology and medicine (96). Moreover, both phage display technology and phages have been utilized to create precise vectors for gene therapy in cancer, aiding in the transmission and expression of beneficial genes within cancer cells. Although the application of high precision, cancer specific nanocarriers as delivery tools for therapeutic genes shows potential in cancer therapy, clinical trials of *in vivo* gene delivery for gene therapy have revealed a notably decreased tumor target transduction (97). Given that the efficiency of gene therapy is strongly dependent on the efficiency of the therapeutic transgene transporter, developing transfer techniques is a critical emphasis for this approach. Among various options, viral vectors have shown great promise (98). Despite being initially viewed as unsuitable for transferring genes to higher organisms, phages have surprisingly offered a new avenue for creating methods to deliver therapeutic genes. They possess several advantages, such as their inability to target mammalian cells, larger cloning capacity, ease of modification, and the utilization of phage display technology, making them a highly promising tool for cancer gene therapy (89). The ease, speed, and low cost of producing phage particles within bacterial cells are well recognized. When it comes to phage therapy for infectious diseases, the systemic administration of phages has proven to be safe and successful (99). However, for a long time, researchers struggled to effectively utilize these viruses for gene therapy in cancer cells. Phage based gene therapy vectors exhibited significantly limited transgene expression in cancer cells (100–102). In 2006, Hajitou and his team made a significant breakthrough by suggesting a solution. They hypothesized that combining the genomes of prokaryotic and eukaryotic viruses would result in the creation of chimeric viral particles that would benefit from the benefits of both original vectors (103). This innovative approach opened up new possibilities for gene therapy, offering the potential to enhance transgene expression in cancer cells using modified phage vectors. The hybrid virus’s genome was created by introducing a recombinant AAV transgenic cassette into the DNA of the M13 filamentous phage. The transgenic cassette contained the HSVtk gene derived from the herpes simplex virus, regulated by the CMV promoter. Surrounding the cassette were two complete inverted terminal repeats (ITRs) from AAV serotype 2. To achieve target specificity for gene delivery, the pIII protein of the phage capsid exposed the RGD4C peptide This peptide was designed to precisely target the v3 integrin receptor, which is prevalent in tumor cells and their surrounding vascular environment but not in healthy tissue cells (104). As a result, the hybrid virus achieved highly effective and stable tumor specific expression of the HSVtk gene. This gene served two purposes: first, as a reporter gene for molecular PET imaging, and second, as a tumor cell suicide gene when combined with ganciclovir (GCV). The success of this approach was evident in suppressing implanted tumors in laboratory mice and rats (103). Consequently, this versatile chimeric virus has found applications in various cancer gene therapy strategies.

Phages have emerged as promising gene therapy vectors for cancer in numerous studies and clinical trials. While eukaryotic viruses were previously favored due to their higher efficiency in transducing mammalian cells, their natural tropism towards eukaryotic host cells presents challenges for therapeutic applications. Retroviral and lentiviral vectors have drawbacks such as potential oncogenicity, restricted expression of the target transgene, and the significant immunogenicity associated with adenovirus-based vectors. These factors have impeded the progress of gene therapy methods for cancer treatment (105). Recombinant adeno associated virus (AAV) vectors have displayed potential because of their efficacy, but they have limitations like limited packaging capacity, the challenge of neutralizing antibodies, and the necessity to enhance transduction selectivity for systemic administration (106). In contrast, phages possess unique structural and biological characteristics that offer innovative methods for targeted gene delivery to cancer cells. By harnessing these features, researchers are exploring the potential of phages as gene therapy vectors, overcoming the limitations of other viral vectors.

## Vaccines based on phages

Vaccines are widely recognized as one of the most crucial advancements in medicine. Since the smallpox vaccine was developed in 1796, numerous forms of vaccinations have been developed to tackle various disease. However, it is important to acknowledge that certain vaccines have exhibited certain limitations, such as high costs and suboptimal immune responses (107). Vaccines have played a pivotal role in preventing and eradicating numerous infectious diseases that were once widespread and posed significant threats to public health (108). Despite their benefits, some vaccines do face challenges in terms of cost and immune response. The production and distribution of vaccines can be complex and expensive, which can pose obstacles, especially in resource limited settings. The cost of research and development, manufacturing, quality control, and distribution can contribute to the high prices associated with certain vaccines. This can limit accessibility, particularly for marginalized communities or countries with limited healthcare resources (109). In addition, while vaccines generally elicit robust immune responses, there can be instances where the immune response is not as strong as desired. Factors such as individual variation in immune systems, the presence of preexisting immunity, or the characteristics of the pathogen itself can influence the effectiveness of a vaccine. Researchers continually strive to enhance vaccine efficacy by optimizing formulations, adjuvants, and delivery methods (110). It is important to note that ongoing research and developments in vaccine progress aim to address these limitations. Efforts are being made to improve vaccine affordability through collaborations between governments, organizations, and pharmaceutical companies. Additionally, ongoing research is focused on optimizing vaccine formulations and delivery systems to enhance immune responses and efficacy (111). Overall, while vaccines have greatly contributed to global health by preventing and controlling diseases, there is ongoing work to address the challenges of cost and immune response. Continued investments in research and development, coupled with global collaboration, will contribute to the development of more accessible and effective vaccines in the future. Phage based vaccines are an emerging approach for antigen delivery in vaccination strategies. phage have been extensively studied for their potential applications in various fields, including biotechnology and medicine (28). In the context of vaccines, phages can be engineered to deliver specific antigens to the immune system. Antigens are substances that stimulate an immune response, leading to the production of antibodies and the development of immunological memory. Vaccines can educate the immune system to recognize and combat certain diseases such as bacteria or viruses by exposing antigens to the immune system (112). There are several advantages to using phages as a platform for antigen delivery in vaccines: (i) Natural affinity for bacteria, phages have a natural ability to infect and target specific bacteria. By engineering phages to display antigens on their surfaces, they can be designed to specifically target bacteria of interest, enhancing the immune response against those bacteria; (ii) High immunogenicity, phages possess inherent immunogenic properties. They can successfully trigger both innate and adaptive immune responses when utilized as a vaccine delivery mechanism. This can lead to a robust immune reaction against the displayed antigens; (iii) Enhanced stability, phages are highly stable and resistant to environmental conditions, such as temperature and pH changes. This stability makes them attractive candidates for vaccine development, as they can withstand the challenges of storage, transport, and administration; (iv) Ease of engineering, phages can be genetically modified relatively easily. Researchers can insert genes encoding specific antigens into the phage genome, resulting in the display of these antigens on the phage surface. This genetic engineering allows for the precise design and customization of phage-based vaccines; (v) Potential for combination vaccines; phages can display multiple antigens simultaneously. This capability opens up the possibility of creating combination vaccines that target multiple pathogens or strains within a single vaccine formulation. This approach can simplify vaccination schedules and improve overall vaccine coverage.

Vaccine development has extensively utilized a diverse range of lytic and filamentous phages for their advantageous characteristics. Filamentous phages, specifically those from the *Inovirus* family such as M13, fd, and f1 phages, have been favored due to their simple capsids, rod shaped structure, and possession of a single-stranded (ss) DNA genome (112). These phages primarily infect bacteria and have a long-standing history in phage display technology (113). The preference for phages in vaccine research is also driven by the availability of well established components and procedures for manipulating the phages, particularly in the areas of antigen and antibody selection and development (27). Recently, an expanded range of phages, including tailed phages like T4, T7, and λ, as well as icosahedral phages like Qβ and MS2, have been employed in phage display vaccine platforms to showcase antigens (114). Unlike filamentous phages, tailed phages offer the ability to express larger peptides and proteins with more complex conformations (115). Filamentous phages, on the other hand, are primarily limited to displaying short peptides (116). This versatility of tailed phages allows for the presentation of a wider range of antigens. Phage display is a technique that involves presenting foreign peptides on the surface of phages. These peptides are fused with a phage coat protein and displayed directly on the phage surface. This approach connects the genotype (the DNA sequence of the displayed peptide) with the phenotype (the displayed peptide), enabling easier testing and selection of peptide antigens. Alternatively, helper phage based systems can be used to display larger proteins on filamentous phages. Due to its speed, cost effectiveness, reliability, and high throughput capabilities, phage display has become a vital tool in vaccine design. It has been widely utilized in the development of vaccines against various microorganisms, including cancer causing pathogens (112). Researchers have employed phage display libraries to identify immunodominant and immunogenic B-cell epitopes that neutralize toxins from pathogens such as *Staphylococcus aureus* (117), *Escherichia coli* (112), *Salmonella typhi* (118), *Borrelia burgdorferi* (119), and viruses like SARS-CoV-2 (120), Ebola Virus (121), and Zika virus (122). Engineered filamentous phages have been employed as direct immunogens in various scenarios, such as immunization against *Plasmodium falciparum*, the parasite responsible for malaria. Initially, the f1 filamentous phage was used as a recombinant immunogen (123). Since then, both filamentous and tailed phages have been used to generate immune responses against viral infections (124–126), parasitic (127), and cancer antigens (128). Extensive pre-clinical *in vivo* studies have examined these approaches. The versatility, affordability, adaptability, and ease of use of phage display make it a powerful tool for discovering potential vaccine candidates and studying antigen-antibody interactions. Its wide range of applications in vaccine development and its ability to target different pathogens highlight its significance in the field. Leveraging phage display allows researchers to efficiently screen and select specific peptide antigens for designing effective immunization strategies against various diseases.

Phages have various applications in the field of DNA vaccines. DNA vaccines entail injecting pure DNA carrying a gene that encodes pathogen antigens. This DNA is controlled by a eukaryotic expression cassette (129). Once inside the cells, the DNA is expressed, leading to antigen production and the induction of an immune response. While DNA vaccines have been found to be effective in mice experiments, their performance in larger animals, nonhuman primates, and human has been mixed (130). Phages offer an alternative method for delivering DNA vaccines. An appropriate eukaryotic promoter is introduced into the phage genome to control the gene expressing the antigen in the phage DNA vaccination system. The DNA is subsequently combined with a eukaryotic expression cassette, which contains critical components such as an open reading frame, a promoter, and a 3’ untranslated region (58). The DNA is packaged *in vitro*, and the resulting vaccine phage is produced by propagating it in *E. coli*. Subsequently, the entire phage is administered to the patient. Antigen presenting cells (APCs) recognize and take up the phages, leading to the expression of the encoded antigen without the need for additional amplification steps (58). This recognition and antigen expression by APCs help initiate an adaptive immune response. Because of their ability to tolerate longer gene sequences, non-filamentous phages such as T4, T7, or phage platforms are commonly used for DNA vaccines. In contrast, Hashemi et al. developed an M13-derived DNA vaccine including an expression cassette for glycoprotein D in HSV-1 which evoked both humoral and cellular immune responses in a mouse model (131).

**Figure 3:**
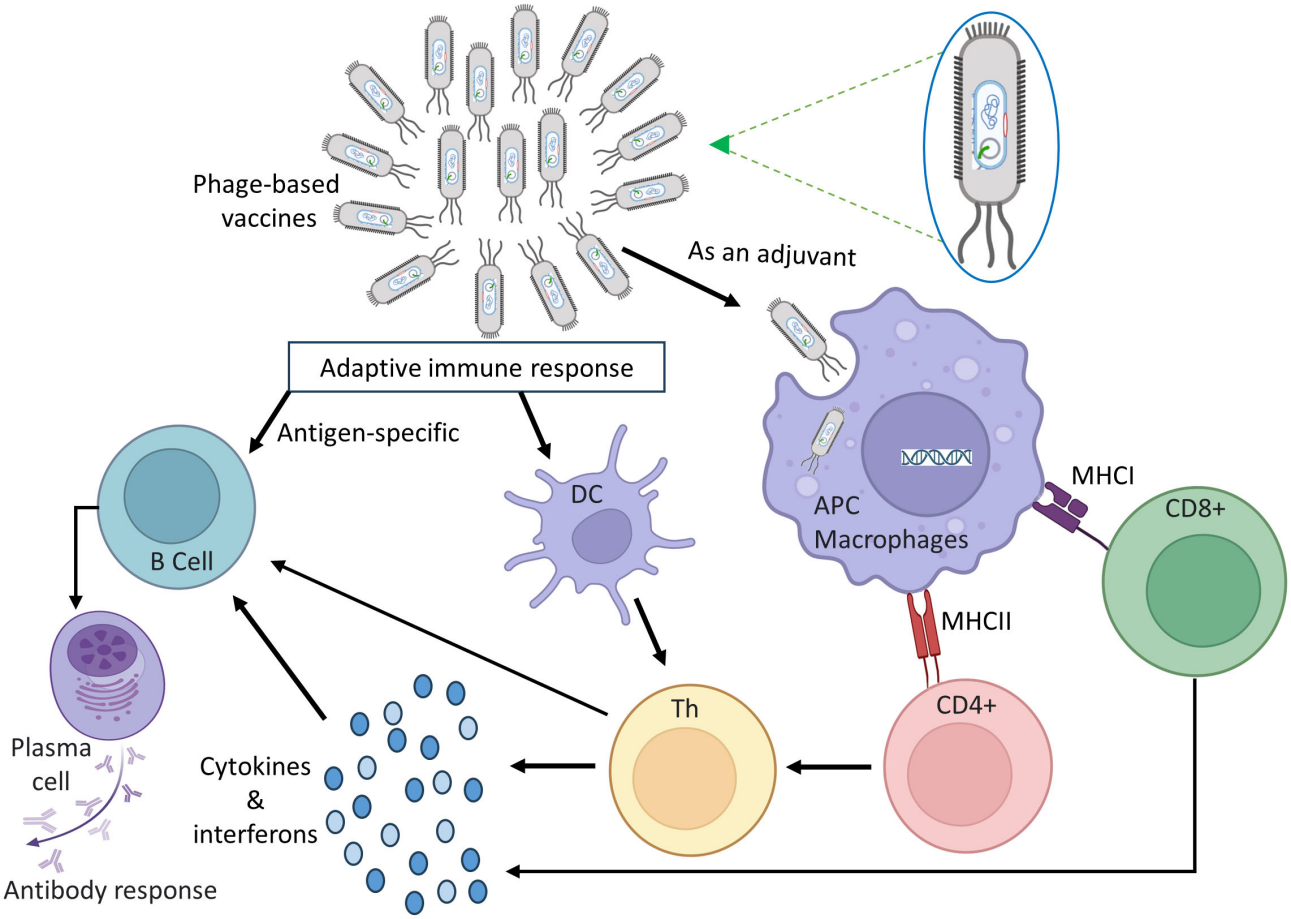
A diagram illustrating the mechanism by which phage vaccines trigger immune responses.

Phage based vaccines are engineered to elicit a durable adaptive immune response, triggering the generation of insistent antibodies, B-cells, and T-cells those specifically recognize the vaccine’s target. **Figure 3** depicts how the immune system’s innate and adaptive components work together to establish immunological memory. Phages activate the innate immune response by interacting with pattern recognition receptors (PRRs) on APC cells such as macrophages and dendritic cells. These phages include genetic information that can activate a variety of viral PRRs, including TLRs 3, 7, 8, 9, and 13 [43, 44]. Phages are considered promising vaccine carriers because they possess a relatively high number of CpG sequences in their genomes, which are recognized by TLR-9 and contribute to their inherent immunogenicity (132). Gogokhia et al. made a discovery that a specific group of lytic phages can induce the production of Type I Interferon in a manner dependent on TLR9 (133). Apart from activating viral PRRs, phages also have the ability to bind to bacterial endotoxins or lipopolysaccharides (LPS) that are released during bacterial lysis (134). LPS is recognized by the PRR and TLR4 of bacteria (135). The natural stimuli aid in the maturation of antigen-presenting cells, enhancing their capacity to effectively present phage peptide to both B-cells and T-cells. Phages play a role in driving antibody responses and humoral immunity, which are critical components of successful vaccination. In one investigation, the residues of the transmembrane protein M2 from the Influenza A virus were fused with the N-terminus of the main coat protein of the M13 phage to generate M2e (2–9) peptide-specific IgG antibodies in broiler chickens (136). Phage viruses also boost cellular immunity. Wang et al. used M13 phage display to discover T-cell and B-cell reactive determinants of *Echinococcus granulosus*, a bacterium that can cause zoonotic illnesses in animals and people (137). They verified the establishment of an antibody response against these epitopes using Western blot and ELISA analysis of sera from rabbits vaccinated with recombinant Eg95 and patients infected with *E. granulosus*, albeit T-cell activation tests were not performed. A M13-derived phage library and sera from patients with tegumentary leishmaniasis were employed in another recent investigation to identify epitopes targeted against *Leishmania amazonensis* infection (138).

## Discussion

Cancer remains a significant global health challenge, resulting in millions of lives lost each year. The limitations of current treatment options, such as side effects, costs, and variable success rates, highlight the urgent need for more targeted and alternative therapies to improve patient outcomes and quality of life. Phages have emerged as promising candidates in cancer therapy due to their high specificity, enabling targeted delivery to cancer cells while sparing healthy ones. This precision provides the opportunity to minimize harmful side effects linked to standard treatments such as chemotherapy and radiation therapy. Furthermore, phages can be genetically modified and personalized, enabling tailored treatments based on individual patients’ unique cancer mutations or genetic abnormalities, potentially enhancing treatment efficacy and reducing resistance risks. Additionally, phages show potential as nanocarriers for delivering therapeutic agents and as vehicles for gene therapy. Their application in vaccine development to stimulate immune responses against cancer cells also holds promise. Continued research into phage-based therapies could revolutionize cancer treatment and bring hope to patients worldwide.

Cancer treatment has advanced significantly, offering various conventional approaches like surgery, chemotherapy, radiation therapy, immunotherapy, targeted therapy, and more. Each has limitations persist, such as side effects, resistance, and cost. Phage therapy has gained attention as a promising alternative due to its specificity, versatility, and genetic modifications, enabling targeted delivery to cancer cells while sparing healthy ones. Its safety profile, potential for reducing toxicity, and ability to penetrate the tumor microenvironment make it an appealing option for future cancer therapies. Personalization based on specific cancer types or mutations enhances its efficacy, and its immune modulating capabilities may further strengthen the body’s cancer fighting ability.

Phage display technology has been instrumental in identifying peptides and antibodies that specifically target cancer cells. By engineering phages to display these ligands, they can be used to deliver cytotoxic agents or induce immune responses, leading to the destruction of cancer cells. Additionally, phages can serve as gene delivery vehicles, offering a site-specific genome integration, a crucial requirement in gene therapy for cancer. Numerous studies have demonstrated the potential of phages in preclinical models, showing significant reductions in tumor size and potential immunotherapeutic benefits (139). Phage therapy holds promise not only as a cancer treatment but also as a tool to protect cancer patients against bacterial infections (15). By leveraging the specificity and safety of phages, they can be used in combination with conventional cancer treatments to improve their efficacy and reduce the risk of infections in immunocompromised patients.

The tumor microenvironment is a complex and dynamic ecosystem that plays a crucial role in tumor growth and progression. The presence of stromal cells, CAFs, angiogenic endothelial cells, and immune cells, as well as the alterations in the ECM and physical and chemical factors, create a unique niche that supports cancer cell survival and expansion. Phages have emerged as promising theragnostic options in the field of cancer research and applications. Their nano size, polyvalent surface properties, non-pathogenic nature, and versatility for chemical or genetic modifications make them attractive for both therapeutic and diagnostic purposes. Phages can be engineered to specifically target cancer cells and penetrate the tumor microenvironment more effectively, enhancing drug delivery to cancerous cells. Their ability to evade clearance by the RES and cross biological barriers, such as the blood brain barrier, further enhances their potential therapeutic efficacy (87). The high surface to volume ratio of phages allows them to efficiently carry large loads of therapeutics to cancer cells, surpassing the capabilities of macromolecular chemotherapeutics. This characteristic makes them valuable tools for innovative drug delivery strategies, potentially reducing off-target effects and improving the overall therapeutic outcome. The versatility of phages enables them to be tailored for personalized therapy, incorporating drugs, targeting ligands, and other therapeutic agents based on individual patient needs. Their ability to carry payloads and deliver them to specific tissues or cells opens up new avenues for targeted therapies and diagnostic applications in the field of cancer.

Phage based cancer gene therapy is a promising and innovative approach in cancer research. It uses phages as gene delivery vehicles to specifically target cancer cells, minimizing side effects. Unlike mammalian viruses, phages offer safety advantages and allow for diverse treatment options. Therapeutic genes delivered by phages can induce cancer cell apoptosis, inhibit tumor growth, or boost the immune response against cancer. The ease of phage manipulation and surface protein engineering facilitates the creation of a library of phage variants for personalized therapy tailored to specific cancer types and individual patients. The development of hybrid virus particles combining prokaryotic and eukaryotic viruses has also shown great potential in enhancing transgene expression for effective cancer gene therapy. Phage based vaccines present a promising and innovative approach to vaccination, addressing challenges related to cost and immune response. Engineered phages can display specific antigens on their surfaces, enhancing targeted immune responses against particular bacteria or viruses. Their high immunogenicity, stability, and ease of engineering make them versatile platforms for vaccine development. Combination vaccines using phages can simultaneously target multiple pathogens, streamlining vaccination schedules and improving coverage. Phage display technology enables efficient screening and selection of peptide antigens, aiding the design of effective immunization strategies against various microbial pathogens. Phage based DNA vaccines show promise in delivering DNA encoding antigens, triggering immune responses without additional amplification steps. This approach holds potential for improving DNA vaccine performance in larger animals and humans, further advancing vaccine research and development. Phage therapy holds promise for cancer treatment, with potential to transform care through more effective and personalized therapies. Phage based vaccines offer a cutting edge approach, overcoming traditional vaccine limitations for targeted and effective cancer immunization. Continued investment in phage-based vaccine research will advance global health and disease prevention.

## Limitations and future trends

Phage based cancer therapy shows great promise as a potentially safe, effective, and personalized treatment option. However, several limitations and challenges must be addressed to fully harness its potential. One significant limitation is the potential for immune responses against the phages, which could reduce their efficacy and limit their use in patients with pre-existing immune dysfunction. To overcome this, further research is needed to understand the mechanisms of phage-induced immune responses and develop strategies to mitigate them, such as using modified phages that are less immunogenic. Another challenge is the development of resistance to phages, which could reduce their long term efficacy. Bacteria can evolve and develop resistance mechanisms against phages, similar to the way they do with antibiotics. Addressing this issue requires continuous monitoring and adaptation of phage formulations to stay ahead of bacterial resistance mechanisms. In addition, optimizing the display of antigens on phage surfaces for use in phage based vaccines is another challenge. This involves finding the most effective way to present cancer specific antigens on phages to stimulate a robust immune response against cancer cells. Ensuring safety and efficacy is also crucial for phage based therapies. Rigorous preclinical and clinical trials are necessary to evaluate the potential side effects and therapeutic benefits of phage treatments accurately. Large scale production of phages is another future trend that needs attention. To make phage therapy widely available, cost effective and scalable production processes must be developed to meet the demand for treatment. Moreover, regulatory approval is a critical hurdle that phage based therapies must overcome. Collaborations between scientists, clinicians, and industry stakeholders are essential to streamline the regulatory process and demonstrate the safety and efficacy of these novel treatments. Despite these limitations and challenges, ongoing research and investment in the field of phage based cancer therapy hold the key to unlocking its full potential. Combining the strengths of conventional cancer treatments with the targeted and personalized benefits of phage therapy could lead to more effective, less toxic, and affordable cancer treatment options in the future.

## Author contributions

MSI: Conceptualization, Data curation, Formal Analysis, Investigation, Software, Writing – original draft, Writing – review & editing. JF: Conceptualization, Writing – original draft. FP: Conceptualization, Funding acquisition, Project administration, Resources, Supervision, Visualization, Writing – review & editing.
